# Supplementary figures and images for: Transcriptome Analysis Reveals Association of E-Class AmMADS-Box Genes with Petal Malformation in Antirrhinum majus L
Source: Int J Mol Sci. 2025 May 7;26(9):4450. doi: 10.3390/ijms26094450 (PMC12072684; doi:10.3390/ijms26094450)

**A**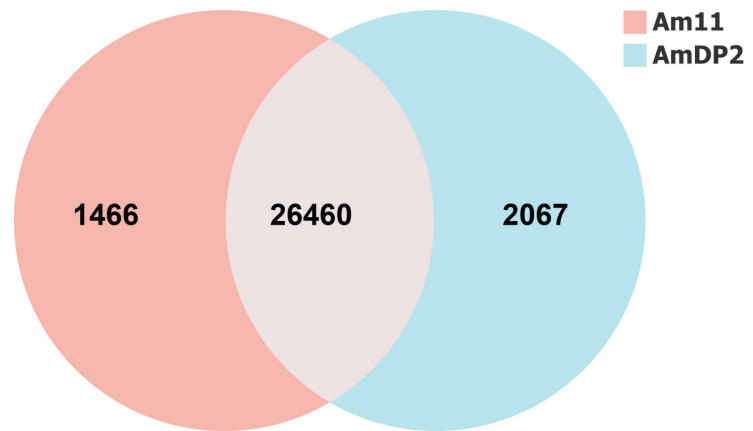**B**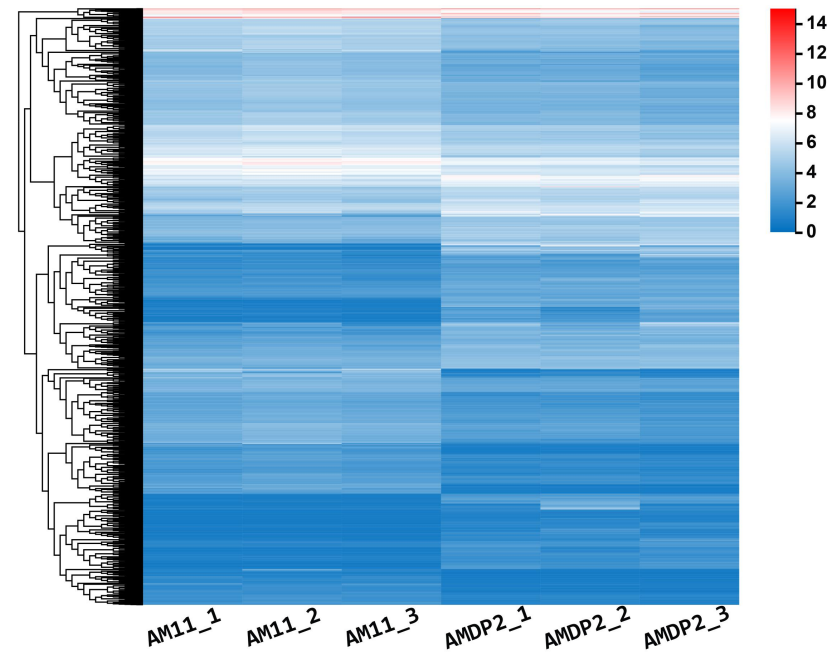**Figure S1**

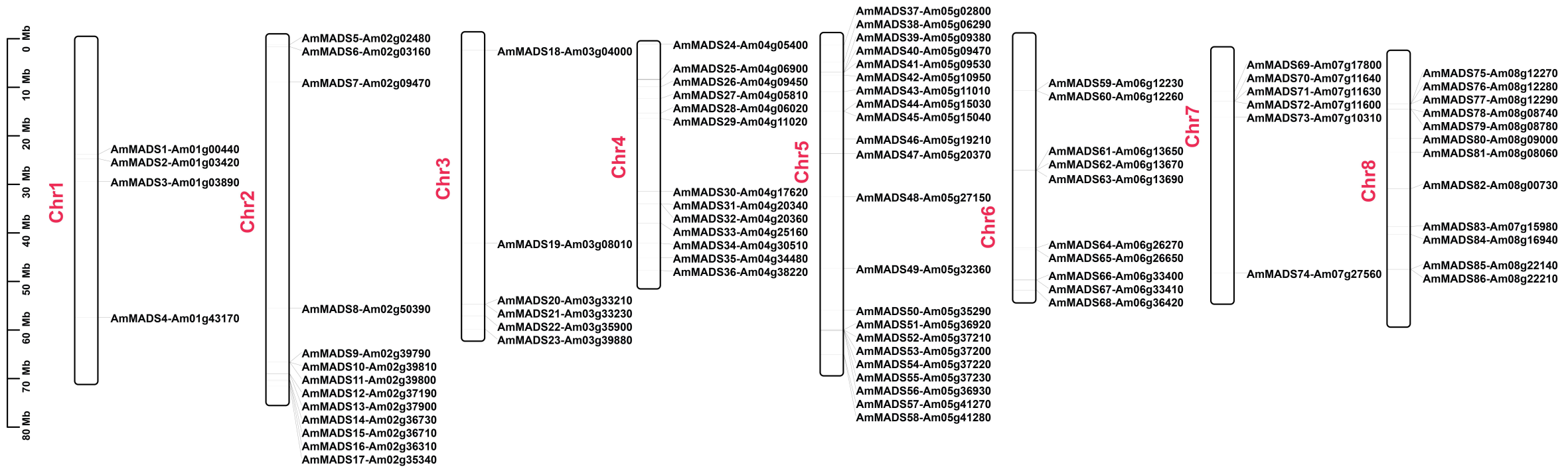

Figure S2

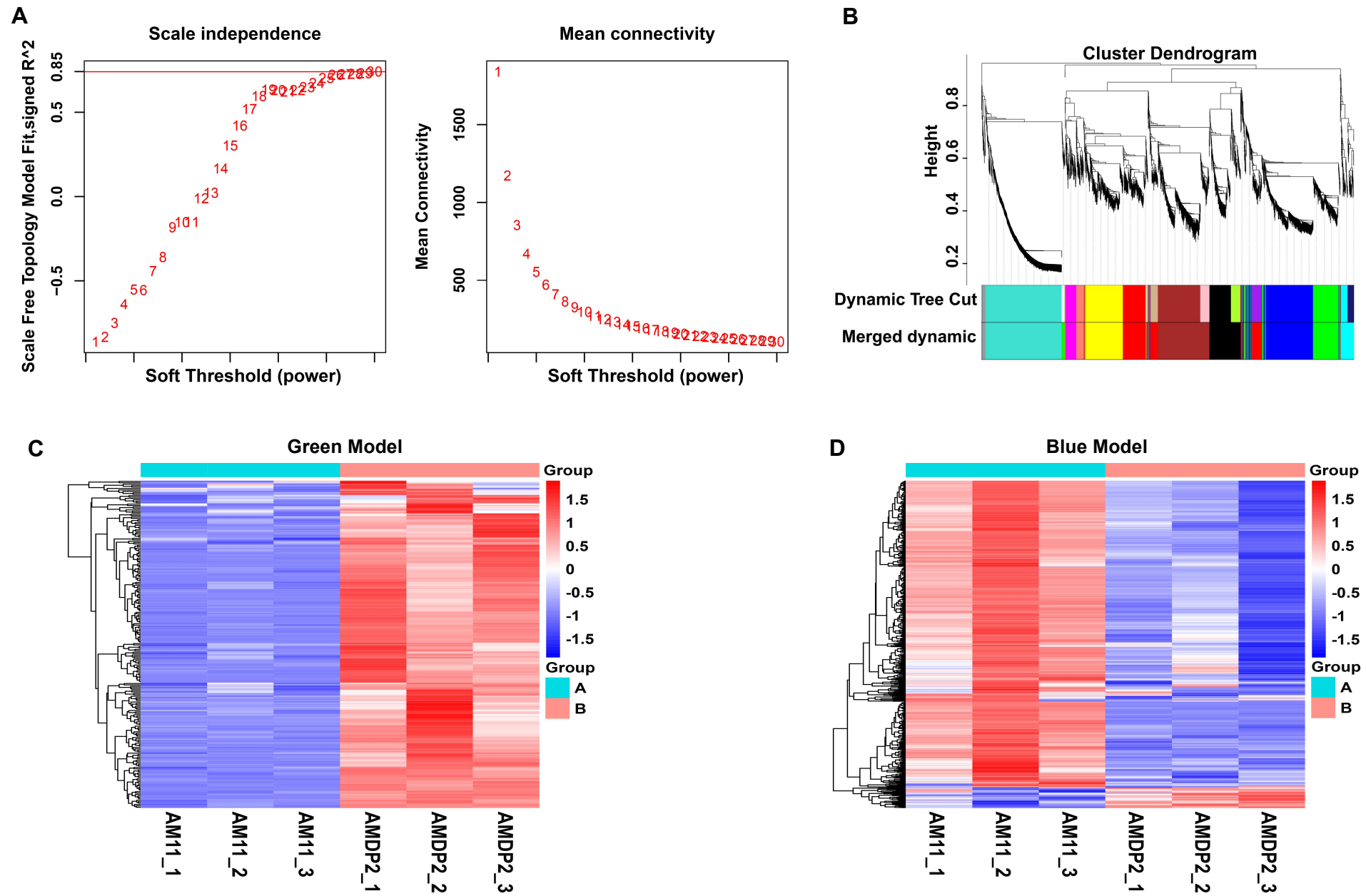

Figure S3

Supplement: Supplementary file 1 [file ijms-26-04450-s001.zip › ijms-3579188-supplementary Figures.pdf]
